# Associations of blood lead levels with reproductive hormone levels in men and postmenopausal women: Results from the SPECT-China Study

**DOI:** 10.1038/srep37809

**Published:** 2016-11-29

**Authors:** Chi Chen, Ningjian Wang, Hualing Zhai, Xiaomin Nie, Honglin Sun, Bing Han, Qin Li, Yi Chen, Jing Cheng, Fangzhen Xia, Li Zhao, Yanjun Zheng, Zhoujun Shen, Yingli Lu

**Affiliations:** 1 Institute and Department of Endocrinology and Metabolism, Shanghai Ninth People’s Hospital, Shanghai JiaoTong University School of Medicine, Shanghai, China; 2 Department of Urology, Huashan Hospital, Fudan University, Shanghai, China

## Abstract

We examined whether blood lead levels (BLLs) were associated with reproductive hormone levels in a cross-sectional study using data from the SPECT-China study. We selected 2286 men and 1571 postmenopausal women without hormone replacement therapy. BLLs, blood cadmium, total testosterone (TT), oestradiol (E2), luteinizing hormone (LH), follicle stimulating hormone (FSH) and sex hormone binding globulin(SHBG) levels were measured. The results showed that median values (interquartile range) of BLLs were 44.00 μg/L (29.00–62.30) for men and 41.00 μg/L (27.00–59.81) for postmenopausal women. In linear regression, after adjusting for age, current smoking status, body mass index, systolic blood pressure, diabetes and blood cadmium level, TT (*P* for trend = 0.001) and SHBG (*P* for trend < 0.001) levels were still positively associated with BLLs in men. Meanwhile, significant positive associations were found for BLLs with SHBG (*P* for trend = 0.002), FSH (*P* for trend = 0.001) and LH (*P* for trend = 0.026) levels in postmenopausal women. Additionally, the association between BLL and SHBG was modified by dysglycaemia (*P* for interaction = 0.03) in postmenopausal women. In conclusion, BLLs were associated with reproductive hormone levels in the general population of Chinese men and postmenopausal women, which may have important implications for human health. Concerted efforts to reduce adult lead exposure are warranted.

Lead is pervasive in the environment and is highly toxic for humans. Although the Chinese government prohibited the use of leaded gasoline in 2000, the rapid rise of China’s lead-acid battery industry^1^, the primitive e-waste recycling procedures^2^, lead-soldered joints of drinking water pipes^3^ and poor air quality^4^ are still major sources of lead pollution. Floating in the air or attaching to objects, lead can be absorbed through ingestion, inhalation, and dermal absorption^5^.

Lead is an environment-disrupting chemical and a reproductive toxin. Even in low concentrations, exposure to lead induced testicular and epididymal toxicity in male Wistar rats^6^. In adult female rhesus monkeys, chronic lead intoxication inhibited the development of follicles and disrupted menstrual cycles^7^. Epidemiological studies exploring the association between blood lead levels (BLLs) and reproductive hormones are limited and inconclusive. Using the data from NHANES 1999–2002, Krieg *et al*.^8^ found that serum FSH and LH levels increased as the blood lead level increased in post-menopausal women, women who had both ovaries removed, and pre-menopausal women. Meeker *et al*.^9^ reported that BLLs in men were not associated with total testosterone (TT), follicle-stimulating hormone (FSH), luteinizing hormone (LH) and sex-hormone binding globulin (SHBG) levels, whereas data from NHANES 1999–2004 showed a positive association between lead exposure and testosterone levels in men^10^.

There has been no study exploring the association of BLLs with reproductive hormones in the general population of Chinese adults. The 2014 Survey on the Prevalence in East China for Metabolic Diseases and Risk Factors (SPECT-China, 2014) measured BLLs in a population from East China. Using these data, we explored the association of BLLs with reproductive hormone levels in Chinese adults. To the best of our knowledge, we are the first to conduct such a large epidemiological study investigating the relationship between blood lead levels (BLLs) and reproductive hormones in China.

## Methods

### Study population

**Figure 1:**
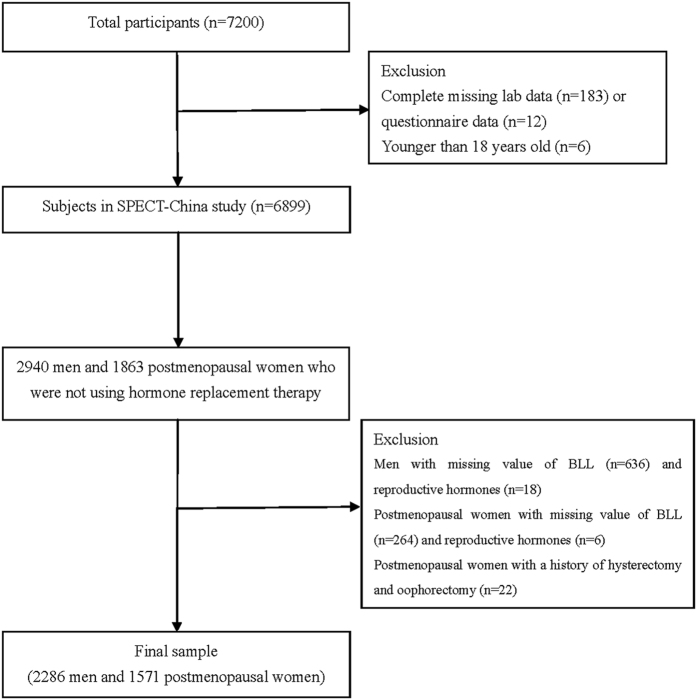
Flowchart of participant’s inclusion and exclusion.

SPECT-China is a population-based cross-sectional survey on the prevalence of metabolic diseases and risk factors in East China. The registration number is ChiCTR-ECS-14005052 (www.chictr.org.cn). Details about the study have been published previously^11^,^12^,^13^. Briefly, from February to June 2014, 16 residential areas in Shanghai, Jiangxi Province and Zhejiang province were selected using a stratified and clustered method. Using the inclusion and exclusion criteria as presented previously, a total of 6899 subjects were enrolled in the SPECT-China study. A woman was considered to be postmenopausal if she was more than 55 years of age^14^,^15^,^16^. In China, approximately 97% of women at the age of 55 are postmenopausal^17^. Initially, there were 2940 men and 1863 postmenopausal women who were not taking hormone replacement therapy. Men with missing values for BLL (n = 636) and reproductive hormones (n = 18) were excluded; women with missing values for BLL (n = 264) and reproductive hormones (n = 6), and with a history of hysterectomy and oophorectomy (n = 22) were also excluded. Finally, the current study was based on a total of 2286 men and 1571 postmenopausal women. An overview of the participants’ inclusion and exclusion in this analysis can be found in the flowchart (Fig. 1).

The study protocol was approved by the Ethics Committee of Shanghai Ninth People’s Hospital, Shanghai Jiao Tong University School of Medicine. All of the following procedures were in accordance with the ethical standards of the responsible committees for human experimentation (institutional and national) and with the Helsinki Declaration of 1975, as revised in 2008. All participants provided written informed consent before data collection.

### Measurements

Data collection was conducted according to a standard protocol by the same trained group of staff at every study site. A questionnaire including information on demographic characteristics, medical history and lifestyle risk factors was completed by the trained personnel. Current smoking was defined as having smoked at least 100 cigarettes in one’s lifetime and currently smoking cigarettes^18^. Weight and height were measured with participants wearing light clothing and no shoes. BMI was calculated as weight in kilograms divided by height in metres squared. Blood pressure was measured following standard methods as described previously^18^. In accordance with the American Diabetes Association 2014 criteria, diabetes was defined as a previous diagnosis by healthcare professionals, fasting plasma glucose (FPG) ≥ 7.0 mmol/L, or glycated haemoglobin(HbA1c)≥6.5%. Dysglycaemia was defined as FPG ≥ 5.6 mmol/L, HbA1c ≥ 5.7%, or a previous diagnosis of diabetes^19^. BMI values were grouped into three categories based on the traditional cut-off ranges: <25 kg/m^2^ was considered ‘underweight and normal’, 25–30 kg/m^2^ was considered ‘overweight’ and >30 kg/m^2^ was considered ‘obese’.

Venous blood samples were drawn from all subjects after an overnight fast of at least 8 h. The blood samples for the plasma glucose tests were collected into vacuum tubes with the anticoagulant sodium fluoride and centrifuged at the site of collection within 1 h after collection. Blood samples were stored at −20 °C after being collected and shipped by air in dry ice to a central laboratory, which was certified by the College of American Pathologists, within 2–4 hours of collection. BLLs and blood cadmium levels were determined via atomic absorption spectrometry (BH2200, China). HbA1c was assessed via high-performance liquid chromatography (MQ-2000PT, China). Total testosterone (TT), oestradiol (E2), luteinizing hormone (LH) and follicle stimulating hormone (FSH) levels were measured using chemiluminescence assays (SIEMENS Immulite 2000, Germany). Sex hormone binding globulin (SHBG) levels were detected using Cobas e601 electrochemiluminescence immunoassays (Roche, Switzerland). The minimum detection limit for each hormone was as follows: 0.7 nmol/L (TT), 73.4 pmol/L (E2), 0.1 IU/L (LH and FSH), and 0.35 nmol/L (SHBG). The intra-assay and inter-assay coefficients of variation for each hormone were as follows: TT, 5.74% and 6.59%; LH, 4.9% and 6%; FSH, 3.82% and 4.48%; E2, 6.24% and 7.52%, respectively, and 7% (both) for SHBG. Samples with values below the minimal detectable limit were given a value midway between zero and the minimal detectable limit for the analyses (0.35 nmol/L for TT and 36.7 pmol/L for E2).

### Statistical Analysis

Analyses were performed using IBM SPSS Statistics, Version 22 (IBM Corporation, Armonk, NY, USA). Two-sided *P* values < 0.05 were considered significant. General characteristics are summarized as median values with the interquartile range (IQR) for continuous variables or as the number with the proportion for categorical variables. To test for differences of characteristics among sex-specific BLL quartiles, Kruskal-Wallis tests were used for non-normally distributed continuous data, and Pearson’s χ2 tests were used for categorical variables. Spearman rank correlations with corresponding significance levels were evaluated to assess the relationships between reproductive hormone levels and BLLs.

The associations of BLLs (independent variable) with reproductive hormones levels (dependent variable) were assessed via linear regression. Model 1 was unadjusted. Model 2 included demographic terms for age and current smoking status. Smoking was adjusted because it is still a substantial source of exposure to lead in general population^20^ and associated with reproductive hormones^21^. Model 3 included the terms for model 2, BMI, systolic blood pressure, diabetes and blood cadmium level. Considering the fact that diabetes and systolic blood pressure have been gradually revealed to be associated with reproductive hormones^16^,^22^, they were adjusted. Blood cadmium was adjusted, because BLLs and blood cadmium levels were significantly correlated in men (Spearman’s correlation coefficient = 0.357; P < 0.001) and postmenopausal women (Spearman’s correlation coefficient = 0.412; P < 0.001) in the present study. SHBG, TT, E2, FSH and LH values were log-transformed because of their skewed distributions. The results were expressed as unstandardized coefficients and standard errors.

Based on the study conducted by Kresovich *et al*.^10^, we further investigated potential interactions of the associations between BLLs and reproductive hormone levels in relation to smoking status, dysglycaemia, BMI, and blood cadmium level. These potential interactions were evaluated based on a cross-product interaction term for the ordinal variable representing BLL quartiles and categorical variables indicating smoking status (current versus former and never combined), glucose status (dysglycaemia versus normal), exposure to cadmium above the median level (median BCL = 1.91 μg/L for men and 1.41 μg/L for postmenopausal women), or BMI (underweight/normal, overweight, and obese)^10^.

Since menopause in our study was based on age, we performed a sensitivity analysis by raising the cut-off age of menopause to 60. Medications taken for diabetes and hypertension may affect the covariates, so we re-performed the regression analyses after excluding subjects taking medications for these conditions.

## Results

### General characteristics of participants

The general characteristics of the study population are summarized in Table 1. This study recruited 2286 men and 1571 postmenopausal women. Medians (IQR) for BLL were 44.00 μg/L (29.00–62.30) for men and 41.00 μg/L (27.00–59.81) for postmenopausal women.

Table 2 shows the characteristics of participants according to BLL quartiles. The quartile ranges for BLL were <29.00, 29.00–43.99, 44.40–62.29, >62.29 μg/L in men and <27.00, 27.00–40.99, 41.00–59.80, >59.80 μg/L in postmenopausal women. For men, compared with the participants in the lowest quartile, those in the highest quartile were older and were more likely to be smokers and have diabetes. These two groups of men had similar E2 levels, but those in the highest quartile had significantly higher TT, FSH, LH and SHBG levels. In postmenopausal women, those in the highest BLL quartile were older and had greater BMI. They also had higher FSH and SHBG levels. However, there was no significant difference across the quartiles for LH, E2 and TT levels in postmenopausal women.

### Correlation between blood lead level and reproductive hormone levels

Correlation results are presented in Table 3. BLLs were significantly and positively correlated with age, systolic blood pressure, TT, SHBG, LH, FSH and blood cadmium levels in men, whereas in postmenopausal women, BLLs were significantly and positively correlated with age, BMI, SHBG, LH, FSH and blood cadmium levels.

### Association of BLL with reproductive hormones assessed via linear regression

Tables 4 and 5 show the results of the linear regression models exploring the associations of the quartiles of blood lead exposure with SHBG, TT, E2, FSH and LH levels in men and postmenopausal women. Using the lowest BLL quartile as a reference (assuming zero change in reproductive hormone levels), significant positive trends were observed for BLLs with SHBG (*P* for trend < 0.001), TT (*P* for trend = 0.012), FSH (*P* for trend < 0.001) and LH (*P* for trend < 0.001) levels in men (Table 4, model 1). After fully adjusting for age, current smoking status, BMI, systolic blood pressure, diabetes and blood cadmium levels, TT and SHBG levels were still positively associated with BLLs (Table 4, model 3). Notably, multivariable analyses revealed a marginal association of BLLs with FSH (*P* for trend = 0.067) and LH (*P* for trend = 0.065) levels in men. There was no association between BLLs and E2 levels in men. However, in postmenopausal women, for all models (Table 5, models 1–3), significant positive associations were found for BLLs with SHBG, FSH and LH levels. There were no associations of BLLs with TT and E2 levels in postmenopausal women.

In relation to the association between BLLs and SHBG levels in postmenopausal women, there was a significant interaction between BLLs and the presence of dysglycaemia in postmenopausal women (*P* for interaction = 0.03). Thus, in the subgroup analysis, there was a significant positive trend among women without dysglycaemia (*P* for trend = 0.003), but was only marginally significant among individuals with prediabetes or diabetes (*P* for trend = 0.088). No other interactions were found between BLLs and other reproductive hormones in relation to smoking, dysglycaemia, BMI or blood cadmium level (all *P* for interactions>0.05) in men and postmenopausal women.

### Sensitivity analysis

In the sensitivity analysis, when we increased the cut-off age of menopause to 60 years, the associations of BLLs with SHBG, FSH and LH levels were still significant (all *P* for trends < 0.05) (Supplementary Table S1). Furthermore, excluding subjects taking medications for diabetes and hypertension did not change the observed associations (all *P* for trends < 0.05) in men (Supplementary Table S2) and postmenopausal women (Supplementary Table S3).

## Discussion

In this population-based study, we observed that BLLs were associated with reproductive hormone levels in Chinese adults without hormone replacement therapy after fully adjusting for age, smoking status, BMI, systolic blood pressure, diabetes and blood cadmium levels. BLLs were associated with higher levels of TT and SHBG in men. A marginal positive association of BLLs with FSH and LH levels was also found in men. Among postmenopausal women, BLLs were positively associated with FSH, LH and SHBG levels. This is the first study to explore the association between BLLs and reproductive hormone levels in Chinese adults.

Only a limited number of epidemiological studies have explored the association between BLLs and reproductive hormones. Our results are consistent with several population-based studies conducted in U.S. men^10^,^23^, Croatian men^24^ and U.S. postmenopausal women^8^,^25^. Nevertheless, Meeker *et al*.^9^ reported the BLLs were not associated with TT and SHBG levels in men. However, the median BLL in Meeker *et al*.’s study was 15.0 μg/L. It is not clear whether different BLLs in different ethnic groups have different effects on male reproductive hormones, and this needs further investigation. Additionally, compared with Meeker et al´s analysis, this study controlled for the same demographic and metabolic covariates, such as age, smoking status and BMI, but also made adjustments for diabetes, systolic blood pressure and blood cadmium levels. Thus, the results from this analysis between BLLs and reproductive hormone levels may be more reliable than the results from previous studies.

We observed marginal and statistically significant positive associations in men and postmenopausal women, respectively, of BLLs with FSH and LH levels. Two possible mechanisms may explain these increases in FSH and LH levels. First, lead can cross the blood-brain barrier^26^ and directly disrupt the hypothalamic-pituitary axis^27^. Long-term low-dose lead exposure has been shown to cause a significant increase in gonadotropin-releasing hormone mRNA in Sprague-Dawley rats^28^, which may stimulate the secretion of FSH and LH. Second, lead could also act indirectly by elevating homocysteine concentrations^29^. A meta-analysis demonstrated that compared with control women, PCOS patients presented higher circulating homocysteine concentrations^30^. In addition, homocysteine serves as an N-methyl-D-aspartate agonist^31^ and a GABA antagonist^32^. N-methyl-D-aspartate stimulates FSH and LH release^33^, and GABA plays a major role in the regulation of gonadotropin-releasing hormone neuron activity and secretion^34^.

Furthermore, we also observed a positive association between BLLs and TT levels in men. There is clear evidence that oxidative stress contributes to lead-associated tissue injury in the testes of male rats^35^. A growing body of literature has shown that lead induces the generation of reactive oxygen species, increases the level of lipid peroxidation, and inhibits the activity of antioxidant enzymes, including glutathione, catalase and superoxide dismutase^6^,^36^.

Most of BLLs in our subjects were in the normal range according to the Centers for Disease Control and Prevention of USA (<100 μg/L)^37^. However, it is acknowledged that endocrine-disrupting chemicals that influence the effect of natural hormones may also have effects at low doses because even small changes in hormone concentrations can have biologically important consequences^38^. Elevated testosterone levels in relation to lead exposure in males may have significant implications since higher testosterone increases the risk of hepatocellular cancer^39^. Meanwhile, increases in FSH and LH levels as a function of the BLL may also have adverse effects on men and postmenopausal women. Benjumin *et al*.^40^ revealed a longitudinal inverse relationship of higher levels of FSH and LH with lower hip bone mineral density, indicating that men with higher levels of FSH and LH lose more bone over time. Saxena *et al*.^41^ reported that in postmenopausal women, elevated levels of LH induced changes in adrenal function towards cortisol secretion, which may contribute to an increased incidence of metabolic syndrome in women after menopause. In addition, it has been revealed that elevated serum FSH levels are independent risk factors for bone loss in postmenopausal women and could be used as indicators in the early diagnosis of postmenopausal osteoporosis^42^.

Notably, we found BLLs to be positively associated with SHBG levels in men and postmenopausal women. To the best of our knowledge, this is the first study to investigate the association between BLLs and SHBG levels in postmenopausal women. SHBG is produced and secreted by the liver into the blood where it binds sex steroids and regulates their bioavailability^43^. Hence, those with higher levels of SHBG may have less bioavailable and albumin-bound testosterone, which is associated with subfertility in men^44^ and atherosclerosis in postmenopausal women^45^. More importantly, SHBG is now regarded as a biomarker of the degree of inflammation in metabolic diseases^43^. Our findings revealed that lead exposure could increase SHBG levels, thus influencing the utility of SHBG as a sensitive biomarker. The mechanism by which lead affects SHBG is unknown, which warrants further investigation.

This analysis also investigated covariates that may modify the association between BLLs and reproductive hormone levels in men and postmenopausal women. We observed a significant interaction for dysglycaemia with BLLs on the association with SHBG levels in postmenopausal women. Recently, using the data of NHANES 1999–2004, Kresovich *et al*.^10^ reported the positive associations between BLLs and testosterone levels could be modified by diabetes, current smoking and blood cadmium in adult men. However, this study recruited non-institutionalized, primarily non-Hispanic American men, and the data may, therefore, not be generalizable to other groups. In addition, compared with our analysis, this study provided no information on postmenopausal women. The detailed mechanism through which dysglycaemia interacts with blood lead remains unknown. Low SHBG is shown to be associated with diabetes in postmenopausal women^46^. It is possible that dysglycaemia and BLLs work antagonistically on SHBG levels, resulting in only minor increases among subjects with diabetes. This may partly explain our results of significantly increased SHBG levels among postmenopausal women without dysglycaemia, while those with prediabetes or diabetes have only marginally increased SHBG levels. Further researches are needed to elucidate potential mechanisms which could explain this finding.

Our study had some strengths. Firstly, this study is novel in that it is the first to investigate the association between BLLs and SHBG levels in postmenopausal women. Secondly, homogeneity and strict quality control of the data are guaranteed by the fact that the same trained staff participated in the survey. Thirdly, our data source was the SPECT-China Study, which was performed in a general population as opposed to a clinic-based population, so our results are more reflective, with detailed information on potentially confounding variables. However, our study also has some limitations. Firstly, the cross-sectional design does not allow us to draw causal relationships between BLLs and reproductive hormone levels. Secondly, we used an age proxy to define the postmenopausal status of women as described in previous studies^14^,^15^,^16^. In China, approximately 97% of women at the age of 55 are postmenopausal^17^. Even we raised the cut-off age to 60, the observed associations did not change in fully adjusted model. Thus, this limitation may not significantly bias the study’s results. Finally, we did not measure albumin, so the calculated free/bioavailable testosterone and estradiol could not be obtained, which is more biologically meaningful. Future studies are warranted to investigate their association in the general Chinese population.

In conclusion, BLLs were associated with reproductive hormone levels in Chinese adults after adjusting for multiple variables. BLLs were positively associated with TT and SHBG levels in men and with FSH, LH and SHBG levels in postmenopausal women. These associations may have significant implications for human health, which warrants further exploration. Concerted efforts to reduce adult lead exposure are warranted.

## Additional Information

**How to cite this article**: Chen, C. *et al*. Associations of blood lead levels with reproductive hormone levels in men and postmenopausal women: Results from the SPECT-China Study. *Sci. Rep.* **6**, 37809; doi: 10.1038/srep37809 (2016).

**Publisher's note:** Springer Nature remains neutral with regard to jurisdictional claims in published maps and institutional affiliations.

## Supplementary Material

Supplementray Tables

## Figures and Tables

**Table 1:** General characteristics of participants.

	Men	Postmenopausal women
Number, n	2286	1571
Age, yr	54 (44–63)	63 (59–68)
BMI, kg/m^2^	24.3 (22.0–26.6)	24.2 (22.1–26.8)
Systolic pressure, mmHg	130 (118–144)	137 (124–152)
TT, nmol/L	15.20 (12.10–19.00)	0.35 (0.35–0.80)
E2, pmol/L	101.50 (36.70–138.00)	36.70 (36.70–91.20)
FSH, IU/l	7.00 (4.80–10.60)	62.70 (48.10–80.70)
LH, IU/l	4.60 (3.30–6.60)	23.10 (17.00–30.00)
SHBG, nmol/L	40.40 (27.90–58.40)	63.40(43.98–89.83)
Blood lead level, μg/L	44.00 (29.00–62.30)	41.00 (27.00–59.81)
Blood cadmium level, μg/L	1.91 (0.60–3.80)	1.42 (0.50–3.30)
Diabetes (%)	13.7	16.9
Current smoker (%)	54.1	4.8

BMI, body mass index; TT, total testosterone; E2, oestradiol; FSH, follicle-stimulating hormone; LH, luteinizing hormone; SHBG, Sex hormone binding globulin.

Data are summarized as median (interquartile range) for continuous variables or as number with proportion for categorical variables.

**Table 2:** Demographic and laboratory characteristics of the study population by blood lead level quartiles.

	Quartile1	Quartile2	Quartile3	Quartile4	P
Men					
Blood lead level, μg/L	<29.00	29.00–43.99	44.00–62.29	>62.29	
Number, n	558	572	585	571	
Age, yr	52 (40–61)	52 (42–62)	55 (44–64)	58 (48–65)	<0.001
BMI, kg/m^2^	24.5 (22.3–26.6)	24.4 (22.0–26.6)	24.2 (21.8–26.6)	23.9 (21.9–26.6)	0.39
Systolic pressure, mmHg	129 (117–141)	128 (116–142)	131 (119–144)	133 (121–148)	<0.001
TT, nmol/L	14.80 (11.90–18.90)	14.90 (12.00–18.40)	15.30 (12.10–18.85)	15.80 (12.60–20.10)	<0.01
E2, pmol/L	98.20 (36.70–135.00)	99.60 (36.70–134.00)	109.00 (36.70–141.50)	101.90 (36.70–141.00)	0.06
FSH, IU/l	6.40 (4.30–9.83)	6.90 (4.50–10.40)	7.00 (5.00–10.25)	7.90 (5.10–11.40)	<0.001
LH, IU/l	4.20 (3.00–6.20)	4.50 (3.20–6.50)	4.70 (3.40–6.45)	4.90 (3.50–7.30)	<0.001
SHBG, nmol/L	36.90 (25.60–54.40)	37.25 (26.10–54.90)	41.40 (28.80–60.05)	44.90 (31.90–62.00)	<0.001
Blood cadmium level, μg/L	0.64 (0.30–2.03)	1.80 (0.60–3.50)	2.40 (0.90–4.20)	3.11 (1.50–4.53)	<0.001
Diabetes,%	14.3	12.1	13.7	14.9	<0.001
Current smoker, %	49	50.1	57.1	60.1	<0.001
Postmenopausal women					
Blood lead level, μg/L	<27.00	27.00–40.99	41.00–59.80	>59.80	
Number, n	382	395	401	393	
Age, yr	62 (58–68)	63 (59–67)	63 (60–68)	64 (60–70)	<0.001
BMI, kg/m^2^	24.2 (22.4–26.6)	23.7 (21.6–26.0)	24.6 (22.6–27.1)	24.7 (22.1–27.7)	0.001
TT, nmol/L	0.35 (0.35–0.80)	0.35 (0.35–0.70)	0.35 (0.35–0.80)	0.35 (0.35–0.90)	0.06
E2, pmol/L	36.70 (36.70–91.78)	36.70 (36.70–95.60)	36.70 (36.70–91.00)	36.70 (36.70–87.50)	0.34
FSH, IU/l	59.10 (45.10–77.00)	63.30 (48.30–80.40)	63.30 (49.25–80.50)	65.10 (49.45–83.95)	0.006
LH, IU/l	21.75 (16.08–29.03)	23.40 (17.30–30.20)	23.10 (17.70–30.95)	23.70 (16.85–30.65)	0.06
SHBG, nmol/L	61.60 (41.70–83.30)	61.85 (44.15–89.15)	61.90 (42.80–89.43)	68.30 (46.40–98.10)	0.02
Blood cadmium level, μg/L	0.50 (0.30–1.18)	1.15 (0.50–2.73)	2.01 (0.71–3.88)	2.79 (1.33–4.40)	<0.001
Diabetes, %	16.2	16.7	14	20.6	0.02
Current smoker, %	4.5	5.6	4.1	5.1	0.8

BMI, body mass index; TT, total testosterone; E2, oestradiol; FSH, follicle-stimulating hormone; LH, luteinizing hormone; SHBG, Sex hormone binding globulin.

Data were summarized as median (interquartile range) for continuous variables or as number with proportion for categorical variables. Kruskal–Wallis test was used for continuous variables and Pearson chi-squared test for categorical variables.

**Table 3:** Spearman correlation coefficients between blood lead level and measured parameters.

Factors	blood lead level, μg/L	
	Men	Postmenopausal women
Age, yr	0.148^***^	0.120^***^
BMI, kg/m^2^	−0.034	0.058^*^
Systolic pressure, mmHg	0.106^***^	0.039
TT, nmol/L	0.062^**^	0.033
SHBG, nmol/L	0.124^***^	0.077^**^
E2, pmol/L	0.036	−0.044
LH, IU/l	0.101^***^	0.054^*^
FSH, IU/l	0.116^***^	0.080^**^
Blood cadmium level, μg/L	0.357^***^	0.412^***^

Data were spearman correlation coefficients. *P < 0.05; **P < 0.01; ***P < 0.001.

BMI, body mass index; TT, total testosterone; E2, oestradiol; FSH, follicle-stimulating hormone; LH, luteinizing hormone; SHBG, Sex hormone binding globulin.

**Table 4:** Association of blood lead level with reproductive hormones in men: linear regression.

	blood lead level, ug/L				P for trend
	Q1	Q2	Q3	Q4	
SHBG					
Model 1	Ref.	0.001 (0.014)	0.039 (0.014)^**^	0.066 (0.014)^***^	<0.001
Model 2	Ref.	−0.003 (0.012)	0.014 (0.012)	0.028 (0.013)^*^	0.01
Model 3	Ref.	<0.001 (0.011)	0.021 (0.011)	0.038 (0.012)^**^	<0.001
TT					
Model 1	Ref.	−0.008 (0.010)	0.004 (0.010)	0.023 (0.010)^*^	0.012
Model 2	Ref.	−0.001 (0.010)	0.002 (0.010)	0.025(0.010)^*^	0.017
Model 3	Ref.	0.001 (0.010)	0.010 (0.010)	0.033 (0.010)^**^	0.001
E2					
Model 1	Ref.	−0.002 (0.016)	0.029 (0.016)	0.016 (0.016)	0.113
Model 2	Ref.	−0.007 (0.016)	0.013 (0.016)	−0.004 (0.016)	0.847
Model 3	Ref.	−0.008 (0.016)	0.014 (0.017)	−0.003 (0.017)	0.794
FSH					
Model 1	Ref.	0.024 (0.016)	0.044 (0.016)^**^	0.088 (0.016)^***^	<0.001
Model 2	Ref.	0.009 (0.014)	0.007 (0.014)	0.031 (0.014)^*^	0.041
Model 3	Ref.	0.010 (0.014)	0.004(0.014)	0.030 (0.015)^*^	0.067
LH					
Model 1	Ref.	0.024 (0.014)	0.041 (0.014)^**^	0.065 (0.014)^***^	<0.001
Model 2	Ref.	0.017 (0.013)	0.013 (0.013)	0.026 (0.013)	0.068
Model 3	Ref.	0.018 (0.013)	0.015 (0.013)	0.028 (0.013)^*^	0.065

Since SHBG, TT, E2, FSH and LH were non-normally distributed, they were log-transformed.

Data were expressed as B coefficients (standard errors). *P < 0.05; **P < 0.01; ***P < 0.001.

TT, total testosterone; E2, oestradiol; FSH, follicle-stimulating hormone; LH, luteinizing hormone; SHBG, Sex hormone binding globulin.

Model 1 was unadjusted. Model 2 included terms for age and current smoking status. Model 3 included the terms for model 2, BMI, systolic blood pressure, diabetes and blood cadmium level.

**Table 5:** Association of blood lead level with reproductive hormones in postmenopausal women: linear regression.

	blood lead level, ug/L				P for trend
	Q1	Q2	Q3	Q4	
SHBG					
Model 1	Ref.	0.021 (0.017)	0.018 (0.016)	0.048 (0.017)^**^	0.007
Model 2	Ref.	0.023 (0.017)	0.009 (0.017)	0.040 (0.017)^*^	0.047
Model 3	Ref.	0.010 (0.015)	0.018 (0.015)	0.048 (0.016)^**^	0.002
TT					
Model 1	Ref.	−0.035 (0.019)	<0.001 (0.019)	0.001 (0.019)	0.504
Model 2	Ref.	−0.038 (0.019)^*^	−0.004 (0.019)	0.005 (0.019)	0.417
Model 3	Ref.	−0.033 (0.019)	−0.017 (0.019)	−0.016 (0.020)	0.612
E2					
Model 1	Ref.	<0.001 (0.019)	−0.019 (0.018)	−0.023 (0.019)	0.126
Model 2	Ref.	−0.004 (0.019)	−0.015 (0.019)	−0.020 (0.019)	0.245
Model 3	Ref.	−0.001 (0.019)	−0.020 (0.019)	−0.021 (0.020)	0.201
FSH					
Model 1	Ref.	0.021 (0.014)	0.037 (0.014)^*^	0.040 (0.014)^**^	0.003
Model 2	Ref.	0.016 (0.015)	0.035 (0.015)^*^	0.035 (0.015)^*^	0.010
Model 3	Ref.	0.013 (0.015)	0.047 (0.015)^**^	0.046 (0.016)^**^	0.001
LH					
Model 1	Ref.	0.030 (0.015)^*^	0.029 (0.015)	0.035 (0.015)^*^	0.031
Model 2	Ref.	0.027 (0.016)	0.024 (0.015)	0.034 (0.016)^*^	0.042
Model 3	Ref.	0.022(0.015)	0.027 (0.016)	0.037 (0.016)^*^	0.026

Since SHBG, TT, E2, FSH and LH were non-normally distributed, they were log-transformed.

Data were expressed as B coefficients (standard errors). *P < 0.05; **P < 0.01; ***P < 0.001.

TT, total testosterone; E2, oestradiol; FSH, follicle-stimulating hormone; LH, luteinizing hormone; SHBG, Sex hormone binding globulin.

Model 1 was unadjusted. Model 2 included terms for age and current smoking status. Model 3 included the terms for model 2, BMI, systolic blood pressure, diabetes and blood cadmium level.
