# Supplementary material for: Associations of blood lead levels with reproductive hormone levels in men and postmenopausal women: Results from the SPECT-China Study
Source: Sci Rep. 2016 Nov 29;6:37809. doi: 10.1038/srep37809 (PMC5127181; doi:10.1038/srep37809)
Supplement: Supplementray Tables [file srep37809-s1.pdf]

Associations of blood lead levels with reproductive hormone levels in men and postmenopausal women: Results from the SPECT-China Study

Running title: lead exposure and reproductive hormones

Chi Chen, Ningjian Wang, Hualing Zhai, Xiaomin Nie, HonglinSun, Bing Han, Qin Li,Yi Chen, Jing Cheng, Fangzhen Xia, Li Zhao, Yanjun Zheng, Yingli Lu

Institute and Department of Endocrinology and Metabolism, Shanghai Ninth People's Hospital, Shanghai JiaoTong University School of Medicine, Shanghai, China

Supplementary Table S1 Association of blood lead level with reproductive hormones in women over 60 years old: linear regression

	blood lead level, ug/L				P for trend
	Q1	Q2	Q3	Q4	
SHBG					
Model 1	Ref.	0.014 (0.019)	0.036 (0.019)	0.042 (0.019) *	0.016
Model 2	Ref.	0.027 (0.020)	0.034 (0.019)	0.045 (0.020) *	0.020
Model 3	Ref.	0.024 (0.017)	0.040 (0.017) *	0.058 (0.018) **	0.001
TT					
Model 1	Ref.	-0.053 (0.022) *	-0.020 (0.022)	-0.015 (0.022)	0.841
Model 2	Ref.	-0.061 (0.023) **	-0.026 (0.023)	-0.014 (0.023)	0.869
Model 3	Ref.	-0.057 (0.022) *	-0.033 (0.023)	-0.031 (0.024)	0.312
E2					
Model 1	Ref.	-0.013 (0.022)	-0.019 (0.021)	-0.023 (0.021)	0.123
Model 2	Ref.	-0.007 (0.022)	-0.025 (0.022)	-0.029 (0.022)	0.212
Model 3	Ref.	-0.011 (0.022)	-0.021 (0.022)	-0.031 (0.023)	0.204
FSH					
Model 1	Ref.	0.036 (0.015) *	0.054 (0.015) **	0.063 (0.015) ***	<0.001
Model 2	Ref.	0.035 (0.016) *	0.054 (0.016) **	0.060 (0.016) ***	<0.001
Model 3	Ref.	0.032 (0.015) *	0.060 (0.016) ***	0.068 (0.016) ***	<0.001
LH					
Model 1	Ref.	0.041 (0.018) *	0.031 (0.018)	0.044 (0.018) *	0.026
Model 2	Ref.	0.042 (0.019) *	0.024 (0.018)	0.046 (0.019) *	0.036
Model 3	Ref.	0.038 (0.019) *	0.027 (0.016)	0.037 (0.016) *	0.033

Since SHBG, TT, E2, FSH and LH were non-normally distributed, they were log-transformed.

Data were expressed as B coefficients (standard errors). $^{*}P < 0.05$; $^{**}P < 0.01$; $^{***}P < 0.001$.

TT, total testosterone; E2, oestradiol; FSH, follicle-stimulating hormone; LH, luteinizing hormone; SHBG, Sex hormone binding globulin.

Model 1 was unadjusted. Model 2 included terms for age and current smoking status. Model 3 included the terms for model 2, BMI, systolic blood pressure, diabetes and blood cadmium level.

Supplementary Table S2 Association of blood lead level with reproductive hormones in men without taking medications for diabetes and hypertension: linear regression

	blood lead level, ug/L				P for trend
	Q1	Q2	Q3	Q4	
SHBG					
Model 1	Ref.	0.009 (0.015)	0.040 (0.015)**	0.070 (0.015)***	<0.001
Model 2	Ref.	0.002 (0.014)	0.013 (0.014)	0.038 (0.014)**	0.004
Model 3	Ref.	0.005 (0.013)	0.023 (0.013)	0.049 (0.014)***	<0.001
TT					
Model 1	Ref.	-0.003 (0.011)	0.011 (0.011)	0.026 (0.011)*	0.009
Model 2	Ref.	-0.001 (0.011)	0.005 (0.011)	0.025 (0.011)*	0.011
Model 3	Ref.	0.001 (0.010)	0.010 (0.011)	0.027 (0.011)*	0.007
E2					
Model 1	Ref.	<0.001 (0.017)	0.032 (0.017)	0.015 (0.017)	0.156
Model 2	Ref.	-0.010 (0.017)	0.012 (0.017)	-0.008 (0.017)	0.988
Model 3	Ref.	-0.011 (0.017)	0.012 (0.018)	-0.007 (0.018)	0.979
FSH					
Model 1	Ref.	0.042 (0.017)*	0.053 (0.017)**	0.101 (0.017)***	<0.001
Model 2	Ref.	0.014 (0.014)	0.006 (0.015)	0.035 (0.015)*	0.033
Model 3	Ref.	0.013 (0.015)	0.002 (0.015)	0.035 (0.015)*	0.054
LH					
Model 1	Ref.	0.042 (0.015)**	0.054 (0.015)***	0.080 (0.015)***	<0.001
Model 2	Ref.	0.027 (0.013)*	0.019 (0.013)	0.035 (0.014)**	0.022
Model 3	Ref.	0.026 (0.013)*	0.019 (0.014)	0.037 (0.014)**	0.022

Since SHBG, TT, E2, FSH and LH were non-normally distributed, they were log-transformed.

Data were expressed as B coefficients (standard errors). $^{*}P < 0.05$; $^{**}P < 0.01$; $^{***}P < 0.001$.

TT, total testosterone; E2, oestradiol; FSH, follicle-stimulating hormone; LH, luteinizing hormone; SHBG, Sex hormone binding globulin.

Model 1 was unadjusted. Model 2 included terms for age and current smoking status. Model 3 included the terms for model 2, BMI, systolic blood pressure, diabetes and blood cadmium level.

Supplementary Table S3 Association of blood lead level with reproductive hormones in postmenopausal women without taking medications for diabetes and hypertension: linear regression

	blood lead level, ug/L				P for trend
	Q1	Q2	Q3	Q4	
SHBG					
Model 1	Ref.	0.014 (0.017)	0.015 (0.017)	0.047 (0.017) **	0.009
Model 2	Ref.	0.024 (0.018)	0.011 (0.018)	0.044 (0.018) *	0.028
Model 3	Ref.	0.011 (0.017)	0.015 (0.017)	0.048 (0.018) **	0.007
TT					
Model 1	Ref.	-0.024 (0.020)	0.021 (0.020)	0.015 (0.020)	0.155
Model 2	Ref.	-0.029 (0.020)	0.020 (0.020)	0.018 (0.021)	0.119
Model 3	Ref.	-0.024 (0.020)	0.005 (0.020)	-0.003 (0.021)	0.768
E2					
Model 1	Ref.	-0.016 (0.021)	-0.027 (0.021)	-0.026 (0.021)	0.180
Model 2	Ref.	-0.019 (0.022)	-0.021 (0.021)	-0.019 (0.022)	0.384
Model 3	Ref.	-0.017 (0.022)	-0.026 (0.022)	-0.022 (0.023)	0.291
FSH					
Model 1	Ref.	0.024 (0.015)	0.032 (0.015) *	0.039 (0.015) *	0.009
Model 2	Ref.	0.021 (0.016)	0.033 (0.016) *	0.036 (0.016) *	0.018
Model 3	Ref.	0.015 (0.015)	0.043 (0.016) **	0.042 (0.016) **	0.002
LH					
Model 1	Ref.	0.034 (0.017) *	0.023 (0.017)	0.040 (0.017) *	0.039
Model 2	Ref.	0.032 (0.017)	0.021 (0.017)	0.043 (0.017) *	0.034
Model 3	Ref.	0.030 (0.017)	0.027 (0.018)	0.048 (0.018) **	0.014

Since SHBG, TT, E2, FSH and LH were non-normally distributed, they were log-transformed.

Data were expressed as B coefficients (standard errors). $^{*}P< 0.05$; $^{**}P < 0.01$; $^{***}P < 0.001$.

TT, total testosterone; E2, oestradiol; FSH, follicle-stimulating hormone; LH, luteinizing hormone; SHBG, Sex hormone binding globulin.

Model 1 was unadjusted. Model 2 included terms for age and current smoking status. Model 3 included the terms for model 2, BMI, systolic blood pressure, diabetes and blood cadmium level.
